# The Role of Visceral Obesity, Sarcopenia and Sarcopenic Obesity on Surgical Outcomes After Liver Resections for Colorectal Metastases

**DOI:** 10.1007/s00268-021-06073-9

**Published:** 2021-04-11

**Authors:** M. Runkel, T. D. Diallo, S. A. Lang, F. Bamberg, M. Benndorf, S. Fichtner-Feigl

**Affiliations:** 1 Department of General - and Visceral Surgery, Medical Center-University of Freiburg, Hugstetterstrasse 55, 79106 Freiburg, Germany; 2 Department of Diagnostic and Interventional Radiology, Medical Center-University of Freiburg, Hugstetterstrasse 55, 79106 Freiburg, Germany

## Abstract

**Background:**

The impact of body compositions on surgical results is controversially discussed. This study examined whether visceral obesity, sarcopenia or sarcopenic obesity influence the outcome after hepatic resections of synchronous colorectal liver metastases.

**Methods:**

Ninety-four consecutive patients with primary hepatic resections of synchronous colorectal metastases were identified from a single center database between January 2013 and August 2018. Patient characteristics and 30-day morbidity were retrospectively analyzed. Body fat and skeletal muscle were calculated by planimetry from single-slice CT images at the level of L3.

**Results:**

Fifty-nine patients (62.8%) underwent minor hepatectomies, and 35 patients underwent major resections (37.2%). Postoperative complications occurred in 60 patients (62.8%) including 35 patients with major complications (Clavien–Dindo grade III–V). The mortality was nil at 30 days and 2.1% at 90 days. The body mass index showed no influence on postoperative outcomes (*p* = 1.0). Visceral obesity was found in 66 patients (70.2%) and was significantly associated with overall and major complication rates (*p* = .002, *p* = .012, respectively). Sarcopenia was observed in 34 patients (36.2%) without a significant impact on morbidity (*p* = .461), however, with longer hospital stay. Sarcopenic obesity was found in 18 patients (19.1%) and was significantly associated with postoperative complications (*p* = .014). Visceral obesity, sarcopenia and sarcopenic obesity were all identified as significant risk factors for overall postoperative complications.

**Conclusion:**

Visceral obesity, sarcopenic obesity and sarcopenia are independent risk factors for overall complications after resections of CRLM. Early recognition of extremes in body compositions could prompt to perioperative interventions and thus improve postoperative outcomes.

## Introduction

Several aspects of body composition, in particular the amount and distribution of body fat and the amount and composition of lean muscle mass, are now understood to be important health outcomes. Obesity is an umbrella term that encompasses the abnormal growth of both visceral (VAT) and subcutaneous adipose tissue (SAT). VAT has greater metabolic consequences than SAT [1]. Visceral obesity is linked to insulin resistance, metabolic syndrome and cancer development [1–3]. The progressive loss of muscle mass, strength and function is known as sarcopenia. It was first described by Rosenberg et al. in the aging population, where it increased all-cause mortality [4–7]. Visceral obesity, sarcopenia and the ‘metabolic double burden’ of sarcopenic obesity are considered potential risk factors for postoperative outcomes [8–15]. However, despite the growing body of literature, the clinical relevance is still inconclusive. This study uses computed tomography-based analysis to examine the role of body composition in the postoperative course after major abdominal surgery. We selected the group of patients with resections of synchronous colorectal liver metastases (CRLM) because of an expected high prevalence of pathological body compositions, a high postoperative morbidity and the availability of preoperative CT scans of the abdomen.

## Methods

The prospective database of our tertiary center was retrospectively searched for patients with first time hepatectomies for synchronous colorectal liver metastases from January 2012 until August 2018. Synchronicity was defined as detection of liver metastases within a 6-months period after initial tumor diagnosis. Patients with an abdominal CT-scan within three months prior to surgery were included in this study. The database included demographic and basic clinical data including surgical procedure and complications. Additional data were acquired from hospital records and outpatient charts. Liver resections were categorized as minor (atypical or <3 segments) or major (>3 segments, hemi-hepatectomies, in situ splits). The postoperative morbidity was graded according to Clavien–Dindo and classified as minor (I–II) and major (III–V) [16].

### Radiological evaluation

**Fig. 1:**
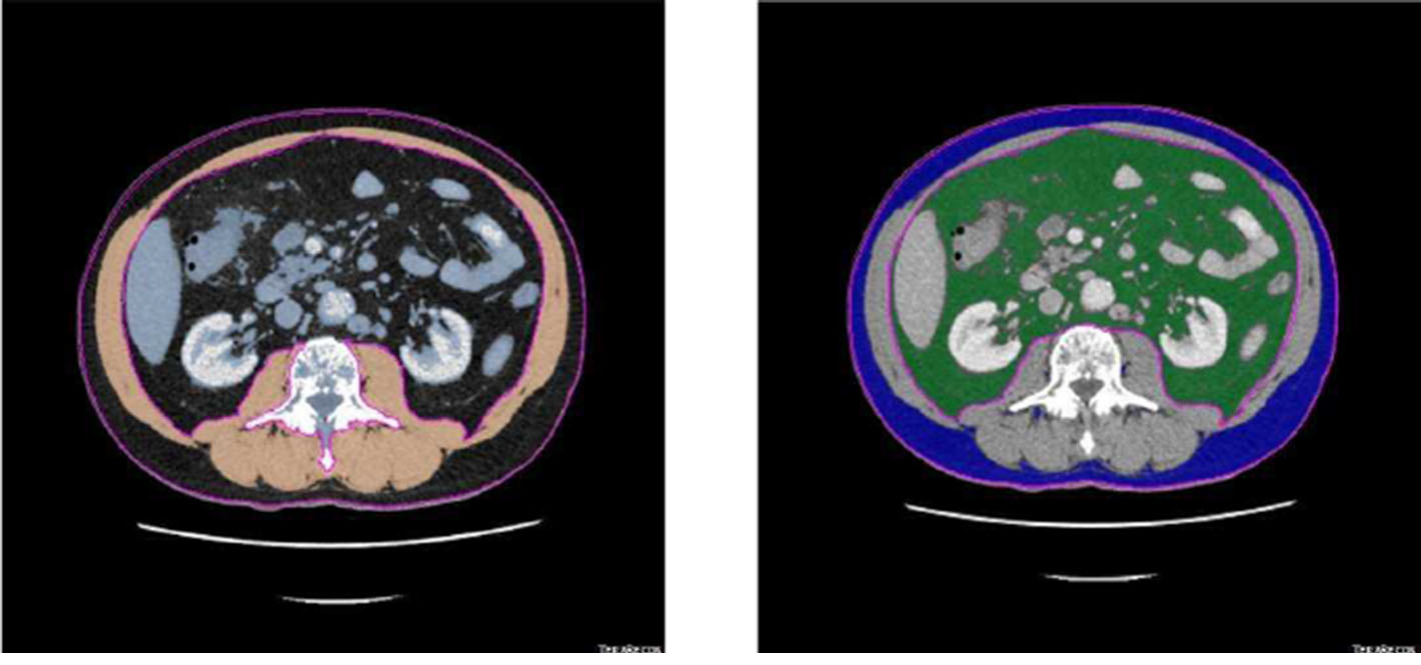
Cross sectional CT imaging at 3rd lumbar vertebra. Muscle tissue is shown in orange color (left), subcutaneous tissue in blue color, and visceral adipose tissue in green color (right)

Skeletal muscle mass, visceral and subcutaneous fat were identified from pre-operative CT scans at the level of L3. Tissue classification was based on Hounsfield units: − 190 to − 30 for subcutaneous fat, − 150 to − 50 for visceral fat and − 29 to 150 for skeletal muscle mass (Fig. 1). The tissue areas were measured semi-automatically using the Aquarius INtuition viewer, Version 4.4, TeraRecon, San Mateo, CA, USA. Body compositions were categorized as normal or pathological according to different definitions found in the literature (Table 1).

**Table 1:** Patients’ characteristics and postoperative outcomes

Total number of patients	94
Age (years)	61.4 years (34–83)
Male	58 (61.7%)
Female	36 (38.3%)
*Neoadjuvant therapy*	
None	35 (37.2%)
preoperative chemotherapy	59 (62.8%)
*Oncologic concept*	
“Liver first” approach	21 (22.3%)
*“Primary first”/simultaneous resection*	
Comorbidities	73 (77.7%)
Arterial hypertension	30 (31.9%)
*Coronary artery disease*	
Diabetes mellitus	16 (17.0%)
Type of operation	13 (13.8%)
Minor	58 (61.7%)
Major	36 (38.3%)
*Postoperative course*	
Regular	34 (36.2%)
Complicated	60 (63.8%)
Minor complications	25 (26.6%)
Major complications	35 (37.2%)
*Clavien–Dindo*	
None	34 (36.2%)
Grade I	2 (2.1%)
Grade II	23 (24.5%)
Grade IIIa	18 (19.1%)
Grade IIIb	10 (10.6%)
Grade IVa	2 (2.1%)
Grade IVb	3 (3.2%)
Grade V	2 (2.1%)

The study was carried out in accordance with the Declaration of Helsinki and approved by the medical ethics committee by the University of Freiburg (Votum reference 49/19).

### Statistics

Values are presented as median and range. IBM SPSS Version 22 was used for statistical analysis. Differences in nominal variables between groups were analyzed using Fisher exact test. Differences in continuous variables between groups were analyzed using the t-test for parametric data sets or the Mann–Whitney *U* test for nonparametric data sets. Multivariate analysis was performed for type of operation, comorbidities and body compositions with multinominal logistic regression analysis. A confidence interval of 95% was taken, and a *p *value < 0.05 was considered statistically significant.

## Results

A total of 94 patients were included for evaluation. Median age was 61.4 years (34–83 years), with 58% of patients being male. Twenty-one patients underwent liver resections as first-step approach (‘liver first’). 62.8% received neoadjuvant chemotherapy (CTx) before proceeding to surgery. Fifty-nine patients had preexisting comorbidities related to obesity (62.8%). Thirty patients suffered from arterial hypertension, 13 patients suffered from type II diabetes and 16 patients from coronary artery disease (Table 2).

**Table 2:** Frequency of obesity, sarcopenia and sarcopenic obesity in our cohort (*n* = 94)

Parameter	Median (range)	‘Normal’ body composition (definition, *n*, %)	Pathological body compositions (definition, *n*, %)	References
BMI (kg/m^2^)	26.0 kg/m^2^ (13.8–45.6)	< 30: 79 (84.0%)	> 30: 15 (16.0%)	
		≤ 100: 28 (29.8%)	≥ 100: 66 (70.2%)	Cakir et al. [17] Watanbe et al. [18] Ishi et al. [19]
VAT (cm^2^)	175.1 cm^2^ (1.0–537.0)	M ≤ 168/W ≤ 80: 58 (61.7%)	M ≥ 168/W ≥ 80: 36 (38.3%)	Van Vugt et al. [20] Doyle et al. [21]
		VAT/SAT: V/S ≤ 0.4: 19 (20.2%)	VAT/SAT: V/S ≥ 0.4: 75 (79.8%)	Clark et al. [22] Ozoya et al. [23]
Skeletal muscle mass (cm^2^/m^2^)	51.4 cm^2^/m^2^ (32.8–79.43)	M > 52.4/W > 38.5: 60 (63.8%)	M ≤ 52.4/W ≤ 38.5: 34 (36.2%)	Lieffers et al. [9] Harimoto et al. [24] Van Vugt et al. [20] Prado et al. [25]
Sarcopenic obesity (VAT cm^2^ + SMI cm^2^/m^2^)	n/a	VAT ≤ 100 cm^2^ + SMI M > 52.4/W > 38.5: 76 (80.9%)	VAT ≥ 100cm^2^ + SMI M ≤ 52.4/W ≤ 38.5: 18 (19.1%)	Nishigori et al. [26]

Fifty-nine patients underwent minor hepatectomy: atypical resection (*n* = 47), mono-segment resection (*n* = 6), bisegment resection (*n* = 6). Major hepatectomies (*n* = 35) included right hepatectomies (*n* = 19), left hepatectomies (*n* = 12), trisegmentectomies (*n* = 3) and in situ splits (*n* = 1).

No major intraoperative complications occurred. The postoperative course was uneventful in 34 patients, whereas 60 patients experienced one or more complications. Complications were graded as minor, when they did not require therapeutic interventions (Clavien–Dindo grades I and II). Minor complications occurred in 25 patients and included prolonged intestinal paralysis, parenteral nutrition, blood transfusions and delirium or wound infections without the need for intervention. Major complications (Clavien–Dindo grades III–V) occurred in 35 patients. Hepatic, renal and respiratory failure requiring prolonged intensive care treatment occurred in 24 patients. Intraabdominal infections including biliary leaks, cholangitis and sepsis required a therapeutic intervention. Nine patients underwent revisional surgery for bowel perforation, anastomotic leaks, biliary leaks and sepsis. Two patients died within 90 days of inpatient stay after a major resection primarily due to liver failure (Clavien–Dindo Grade V, Table 2).

The incidence of obesity in the study group varied widely from 16 to 80% depending on the definitions used. 36% of patients were identified as sarcopenic, and 19.1% of patients were diagnosed with sarcopenic obesity (Table 1).

The development of overall postoperative complications after liver resections for CRLM was significantly associated with patients’ gender and hypertension in univariate analysis (Table 3). Complications also correlated with obesity when defined by VAT ≥ 100 cm^2^, VAT ≥ 168 cm^2^ for men and ≥80 cm^2^ for women or V/S ≥ 0.4 and with sarcopenic obesity. There was no association with obesity, when defined by BMI, or with sarcopenia. Furthermore, neoadjuvant chemotherapy and the extent of liver resection had no influence on the postoperative course. In multivariate analysis, all definitions of obesity, except for BMI, were independent predictors for overall postoperative complications. Sarcopenia and sarcopenic obesity were also independent variables in multivariate analysis.

**Table 3:** Correlation of variables for overall postoperative complications in univariate and multivariate analysis (values in bold-italic indicate significance at *p* < 0.05)

Multivariate analysis variable	No complications (*n*=)	Complications (*n*=)	Univariate analysis	Log regression analysis CI 95% [odds ratio]
VAT ≤ 100 cm^2^ VAT ≥ 100 cm^2^	17 18	11 48	***p = .002***	***p < .001 (OR 1.6, CI 1.3–1.9)***
VAT ≤ 168 cm^2^/80 cm^2^ VAT ≥ 168 cm^2^/80 cm^2^	19 16	17 42	***p = .014***	***p < .001 (OR 1.5, CI 1.2–1.9)***
V/S ≤ 0.4 V/S ≥ 0.4	12 23	7 52	***p = .015***	***p < .001 (OR 1.5, CI 1.2–1.8)***
BMI < 30 kg/m^2^ BMI > 30 kg/m^2^	30 5	49 10	*p* = 1.0	*p* = .225 (OR 1.2, CI 0.9–1.6)
No sarcopenia sarcopenia	24 11	36 23	*p* = .461	***p = .003 (OR 1.4, CI 1.1–1.6)***
No sarcopenic obesity Sarcopenic obesity	33 2	43 16	***p = .014***	***p = .009 (OR 1.4, CI 1.1–1.8)***
Male Female	15 20	43 16	***p = .004***	
Minor hepatectomy Major hepatectomy	22 13	36 23	*p* = .859	*p* = .179 (OR 1.1, CI 0.9–1.2)
Minor hepatectomy + ≥ Va 100cm^2^ Major hepatectomy + ≥ VAT 100 cm^2^	10 8	31 17	*p* = .574	
No art. hypertension Art. hypertension	29 6	35 24	***p = .022***	*p* = .111 (OR 1.2, CI 0.9–1.5)
No CVD Cardiovascular disease	32 3	46 13	*p* = .154	*p* = .696 (OR 0.9, CI 0.7–1.3)
No diabetes mellitus Diabetes mellitus	31 4	50 9	*p* = .761	
No neoadj CTx Neoadj CTx	11 24	24 35	*p* = .370	
No neoadj CTx + sarcopenia Neoadj CTx + sarcop	5 6	8 15	*p* = .709	

The development of major complications significantly correlated with the presence of cardiovascular disease, obesity (definitions VAT ≥ 100 cm^2^ and V/S ≥ 0.4) and sarcopenic obesity in univariate analysis (Table 4). There was no association with obesity, when defined by BMI or VAT ≥ 168 cm^2^/80 cm^2^, or with sarcopenia. VAT ≥ 100 cm^2^ was identified as the only significant predictors for major postoperative complications in logistic regression analysis.

**Table 4:** Correlation of variables for major postoperative complications in univariate and multivariate analysis (values in bold-italic indicate significance at *p* < 0.05)

	None/minor complications (*n* =)	Major complications (*n* =)	Univariate analysis	Log regression analysis (Odds ratio, CI 95%)
VAT ≤ 100 cm^2^ VAT ≥ 100 cm^2^	23 36	5 30	***p = .019***	***p = .004 (OR 1.4, CI 1.1–1.7)***
VAT ≤ 168 cm^2^/80cm^2^ VAT ≥ 168 cm^2^/80 cm^2^	26 33	10 25	*p* = .188	*p* = .095 (OR 1.2, CI 0.8–1.4)
V/S ≤ 0.4 V/S ≥ 0.4	15 44	4 31	*p* = .119	*p* = .072 (OR 1.2, CI 0.9–1.5)
BMI < 30 kg/m^2^ BMI > 30 kg/m^2^	51 8	28 7	*p* = .561	*p* = .190 (1.2, CI 0.9–1.6)
No sarcopenia sarcopenia	39 20	21 14	*p* = .658	*p* = .068 (OR 1.2, CI 0.9–1.5)
No sarcopenic obesity Sarcopenic obesity	52 7	24 11	***p = .029***	*p* = .051 (OR 1.3, CI 1.9–1.7)
Male Female	32 27	26 9	*p* = .079	
Minor hepatectomy Major hepatectomy	36 23	22 13	*p* = .859	*p* = .779 (OR 1.0, CI 0.9–1.2)
Minor hepatectomy + ≥ Vat 100 cm^2^ Major hepatectomy + ≥ VAT 100 cm^2^	22 14	19 11	*p* = .853	
No art. hypertension Art. hypertension	44 15	20 15	*p* = .080	*p* = .670 (OR 1.1, CI 0.8–1.3)
No CVD Cardiovascular disease	54 5	24 11	***p = .009***	*p* = .113 (OR 1.2, CI 0.9–1.7)
No diabetes mellitus Diabetes mellitus	51 8	30 5	*p* = 1.0	
No neoadj CTx Neoadj. CTx	20 39	15 20	*p* = .385	
No neoadj CTx + sarcopenia Neoadj CTx + sarcopeniaenia	12 8	9 5	*p* = 1.0	

### Length of stay

Median length of stay on ICU was 3 days (0–55 days), and median length of overall postoperative hospital stay (LOS) was 12 days (5–91 days). The length of hospital stay was not significantly influenced by gender, comorbidities, preoperative chemotherapy or the extent of surgery. The length of stay was 11 days (5–52) days after minor versus 12 days (8–91) after major hepatectomies (*p* = 0.063). There was no association with body composition except for sarcopenia that significantly prolonged the hospital stay [11 days (5–60) versus 14 days (6–91), *p* = 0.028, Table 5].

**Table 5:** Correlation of variables for length of stay in univariate analysis (values in bold-italic indicate significance at *p* < 0.05)

Variables	Average length of stay (median, days)	Significance
VAT ≤ 100 cm^2^ VAT ≥ 100 cm^2^	11 (5–35) 12 (6–91)	*p* = .169
VAT ≤ 168 cm^2^/80 cm^2^ VAT ≥ 168 cm^2^/80 cm^2^	11 (5–60) 12 (6–91)	*p* = .886
V/S ≤ 0.4 V/S ≥ 0.4	10 (5–35) 12 (6–91)	*p* = .431
BMI < 30 kg/m^2^ BMI > 30 kg/m^2^	12 (5–91) 10 (8–36)	*p* = .593
No sarcopenia Sarcopenia	**11 (5**–**60)** **14 (6**–**91)**	***p = .028***
No sarcopenic obesity Sarcopenic obesity	11 (5–60) 15 (6–91)	*p* = .059
Minor hepatectomy Major hepatectomy	11 (5–52) 12 (8–91)	*p* = .063

## Discussion

Body compositions are increasingly being considered as important risk factors for postoperative outcomes. Using BMI to defined obesity, is still widely used in clinical practice, mainly due to its simplicity of calculation; however, BMI is not the most effective measurement of visceral obesity and does not accurately predict postoperative morbidity, as shown in the literature and in the present study [12, 27, 28]. Abdominal surgeons are more interested in visceral obesity, due to potential increased technical difficulties. In the present study of liver resections, visceral obesity (VAT ≥ 100 cm^2^) was an independent predictor of overall and major complications. However, there are other cut-off values found in the literature and, depending on the cut-off, the prevalence varied. In our study population, prevalence was between 16% (BMI > 30 kg/m^2^) and 80% (V/S ≥ 0.4). The cut-offs have a major impact on statistics and limit the comparison of published data.

Muscle mass is a key parameter of body compositions. The role of sarcopenia in surgery has been evaluated by several meta-analyses in recent years. A significantly higher risk for postoperative complications was found in emergency surgery (RR = 2.07, 4 studies, 734 patients [29]) and in surgery for inflammatory bowel disease (OR = 6.097, 10 studies, 885 patients [30]). The morbidity was also increased after gastrectomy (OR 3.09, 8 studies, 2649 patients [31]) and after colorectal resections (OR 2.71, 2 studies, 518 patients [31] but a higher risk of postoperative complications has not been identified after esophageal (OR 0.81, 8 studies, 1488 patients [32]) and pancreatic resections (13 studies, 3608 patients [33]). All commonly used CT-assessed sarcopenia indexes, such as the skeletal muscle index (SMI), predict the risk of major postoperative complications (RR 1.36, 22 studies, 6656 patients [34]).

There are less data regarding sarcopenic obesity in surgery. A recent meta-analysis consisting of 5 studies showed sarcopenic obesity to be significantly associated with complications after colorectal, gastric and pancreatic cancer surgery [14]. In a retrospective analysis of 805 patients, sarcopenic obesity resulted in increased morbidity and mortality after colorectal surgery [15]. Another study showed an increased risk of wound infection after laparoscopic gastrectomy [26].

The present study examined the impact of body compositions after hepatectomy. The study included patients who underwent resection of synchronous colorectal liver metastases. Visceral obesity, sarcopenia and sarcopenic obesity were independent predictors of complication in multivariate analysis, and visceral obesity was also an independent variable for major complications. There are few published series on this topic in the literature and our data seem to concur.

Peng et al. [10] investigated the incidence and influence of sarcopenia in patients undergoing liver resection for CRLM. Sarcopenia was assessed by measuring total psoas area on CT. The morbidity rate was 23% amongst the entire group of 259 patients. In comparison with their counterparts, sarcopenic patients (*n* = 41, 16%) had an increased risk of postoperative major complications and a longer hospital stay. On multivariate analysis, sarcopenia remained independently associated with an increased risk for complications (OR 3.12).

Contradicting results were published in 2015. Lodewick et al. [35] could not confirm an impact of obesity, sarcopenia and sarcopenic obesity on postoperative morbidity. The study shares several aspects of the methodology with our own. The patients (*n* = 171) were identified from a prospective database at a tertiary cancer center and retrospectively reviewed. Body composition was estimated by CT within 3 months before liver surgery. The patients’ characteristics were comparable to our cohort. Sarcopenia and sarcopenic obesity was more prevalent in their patients as compared to ours (47% vs. 36% and 29% vs. 19%).

Kobayashi et al. [36] assessed the effects of the body compositions after liver resection for hepatocellular carcinoma. CT-morphometry was used for the measurements of body composition, and 465 patients were retrospectively analyzed. The innovative part of this landmark study, according to Molinari [37], was that the authors stratified the study population in four groups according to body composition: normal (*n* = 184), obese (*n* = 219), sarcopenic (*n* = 31), and sarcopenic obese (*n* = 31). The overall morbidity rate was 35% with no significant differences between the 4 groups. However, the major morbidity rates were significantly different between normal body composition (17%), obese (20%), sarcopenic (39%) and sarcopenic obese (32%, *p* = 0.016).

Beradi et al. [38] analyzed a cohort of 234 patients undergoing liver resection for malignant tumors (hepatocellular carcinoma 43%, CRLM 41%). The patients’ characteristics were similar to ours regarding gender (67% male), age (median 66 years), BMI 27 kg/m^2^ and extent of hepatectomy (major 27%). Differences were seen regarding comorbidity (70% including liver cirrhosis) and rate of neoadjuvant chemotherapy (38% vs 63%). The frequency of overall and major complications was lower in the Italian cohort (31% vs. 63% and 8% vs. 37%, respectively). Muscle mass and strength were assessed using the SMI on preoperative CT and the handgrip strength test. A reduced SMI alone did not increase the rate of overall (32%) and major (7%) complications. Reduced muscle mass plus grip strength had little impact on overall (34%) but increased the rate of major complications to 18%. Sarcopenia, portal hypertension, liver cirrhosis and biliary reconstruction were independent risk factors associated with 90-day morbidity.

Last year, Inuho et al. [39] published a retrospective analysis to test the benefits of laparoscopic (204 patients) vs open hepatectomy (100 patients) for CRLM. The diagnosis of obesity was based on measurements of BMI, and the diagnosis of visceral obesity was based on assessments of visceral fat area using CT. Both had an unfavorable effect on outcome in patients who had undergone open surgery, but this negative impact was lost when hepatectomy was performed laparoscopically.

The interpretation of our own data in the light of the above 5 publications must be made with great caution, because study populations were not as homogenous as intended by selecting patients with oncologic liver resection for analysis. The small sample size is prone to type 2 errors**.** There is some heterogeneity regarding the underlying cancer (colorectal, hepatocellular), the oncologic concept (“primary first,” “liver first,” neoadjuvant chemotherapy), the associated hepatopathy (post-chemotherapy, cirrhosis) and the prevalence of pathologic body composition.

Another important limitation concerns methodological issues. Different studies apply different definitions of pathological body compositions, different measurement techniques and different cut-off values. Although CT-measurement has been established as the gold-standard, the level and area of measurement differs across the literature. Peng et al. [10], for example, assessed sarcopenia by measuring the cross-sectional area of the psoas muscles. Most commonly, however, the skeletal muscle mass is measured at the level of the third lumbar vertebra (L3).

In 2019, European Working Group on Sarcopenia in Older People (EWGSOP) revised sarcopenia guidelines and recommended using low muscle mass as well as decreased function (strength or performance) to define sarcopenia [40]. The most used and validated method is the hand grip exam—non-dominant hand grip strength. There is no information of muscle strength in the present paper. In fact, there is only one study [38] that submits this information. Interestingly, loss of muscle mass increased the complication rate only in combination with reduced grip strength. This observation emphasizes the importance of measuring both, muscle quantity and quality.

Another source of confusion is cut-off values in CT planimetry. Sarcopenia was defined as SMI of ≤52.4 cm^2^/m^2^ for men and ≤38.5 cm^2^/m^2^ for women in the present study. Lodewick et al. [35] used thresholds of ≤41 cm^2^/m^2^ in women, ≤43 cm^2^/m^2^ in men with a BMI < 25 kg/m^2^ and <53 cm/m^2^ in men with a BMI > 25 kg/m^2^. Other cut-offs were: 53.5 cm^2^/m^2^ in men and 40.8 cm^2^/m^2^ in women [38] and 40.31 cm^2^/m^2^ in men and 30.88 cm^2^/m^2^ in women [28]. Simonsen et al. identified 22 different definitions for sarcopenia [41]. The literature also finds varying definitions for obesity. Obesity was based on body fat percentages in CT with cut-off values of 44.4% for women and 35.7% for men [35]. The Japanese authors used BMI > 25 kg/m^2^ and visceral fat area of >100 cm^2^ to define obesity [39]. The present study has applied several definitions demonstrating their impact on statistical calculations.

## Conclusion

Overall, our data and most of the published results confirm an association between body composition and complications after liver resection. Our study supports the idea of using body composition measurements for preoperative risk stratification. It remains to be shown that preoperative correction of the parameters of body composition improves the surgical outcome.
